# Diagnostic and Therapeutic Potential of Substance P/NK1 Receptor in Primary Dysmenorrhea: A Pilot Study

**DOI:** 10.1002/hsr2.71744

**Published:** 2026-01-18

**Authors:** Riffat Mehboob, Imran Shahid, Abdullah R. Alzahrani, Shimaa Mohammad Yousof, Hafsa Adnan, Samar N. Ekram, Ahmad Alwazzan, Hisham Nasief, Khalid A. Khadawardi

**Affiliations:** ^1^ Lahore Medical Research Center Lahore Pakistan; ^2^ Rotogen Biotech LLC Bethesda Maryland USA; ^3^ Department of Pharmacology and Toxicology, Faculty of Medicine Umm Al‐Qura University Al‐Abidiyah, P.O.Box 13578 Makkah Saudi Arabia; ^4^ Department of Physiology, Faculty of Medicine King Abdulaziz University Rabigh Saudi Arabia; ^5^ Department of Physiology, Faculty of Medicine Suez Canal University Ismailia Egypt; ^6^ Neuroscience and Geroscience Unit, King Fahad Medical Research Centre King Abdulaziz University Jeddah Saudi Arabia; ^7^ Department of Biological Sciences National University of Medical Sciences Rawalpindi Pakistan; ^8^ Department of Medical Genetics, Faculty of Medicine Umm Al‐Qura University Makkah Saudi Arabia; ^9^ Department of Obstetrics and Gynecology, Faculty of Medicine King Abdulaziz University Jeddah Saudi Arabia; ^10^ Department of Obstetrics and Gynecology, Faculty of Medicine Umm Al‐Qura University Makkah Saudi Arabia

**Keywords:** dexamethasone, dysmenorrhea, gynecology, neurokinin‐1 receptor, substance P

## Abstract

**Background and Aims:**

Primary dysmenorrhea is a prevalent condition that causes significant menstrual pain and discomfort, impacting many women′s daily activities. The Substance P/NK1 receptor (SP/NK1R) pathway has been linked to the pain mechanisms underlying dysmenorrhea, yet its potential as a therapeutic target remains inadequately explored. This study aimed to evaluate the therapeutic potential of NK1R antagonists, specifically dexamethasone and aprepitant, in alleviating the symptoms of primary dysmenorrhea.

**Methods:**

A randomized controlled trial was conducted from March 2024 to December 2024 using a convenience sampling technique. Forty female participants were divided into three phases: Phase 1 (pre‐medication), Phase 2 (NSAIDs), and Phase 3 (dexamethasone + aprepitant). Ten control females without dysmenorrhea were also recruited. The study lasted for three menstrual cycles, with assessments of SP/NK1R levels, visual analogue scores (VAS), and pictorial blood loss. Data were analyzed using Two‐way ANOVA with repeated measures.

**Results:**

The mean age of participants was 25.1 ± 7.6 years, and BMI was 20.47 ± 3.61 kg/m². SP/NK1R levels were significantly elevated in Phase 1 compared to controls. NK1R levels decreased significantly from 15.4 ng/mL in Phase 1 to 8.42 ng/mL in Phase 3. VAS scores decreased by 4.79 units. A linear mixed‐effects model revealed significant reductions in NK1R levels in Phase 2 versus Phase 1 (*p* < 0.001) and Phase 3 versus Phase 1 (*p* = 0.006). VAS scores improved significantly in Phase 3 compared to Phase 2 (*p* = 0.0001 for SP and *p* = 0.0028 for NK1R). Heavy period patients had higher SP levels. Both SP and NK1R levels decreased across phases for normal and heavy period patients.

**Conclusion:**

This pilot study suggests that NK1R antagonists (dexamethasone and aprepitant) may reduce discomfort in dysmenorrhea. Further research with a larger sample size and control for intra‐individual hormone levels is needed.

## 1 Introduction

Dysmenorrhea in women of childbearing age is characterized by excruciating menstrual cramps of uterine origin and is among the most prevalent gynecological conditions [1]. Because many affected individuals do not seek medical care, the condition—despite its high frequency often goes underdiagnosed [2, 3]. Dysmenorrhea is classified as primary or secondary based on its etiology. Primary dysmenorrhea (PD) one of the most common complaints in adolescents and adults is defined as spasmodic, painful lower‐abdominal cramps that begin just before or at the onset of menses in the absence of pelvic pathology [4]. It typically emerges during adolescence, within 6 to 24 months after menarche. The pain usually peaks on the first day of menstruation and may persist for up to 72 h, following a distinct cyclical pattern [5].

Dysmenorrhea is associated with mood swings, lethargy, headaches, nausea, and edema during the menstrual cycle. It can adversely affect personal and social functioning, including mood, sleep, and daily activities. Globally, between 2010 and 2015, reported prevalence varied widely from 94% in Oman to 59.80% in Bangladesh, 34% in Egypt, and 0.90% in Korea [6]. A comprehensive review by Kharaghani and Damghanian reported that 71% of Iranian women experience PD [7]. The principal pathophysiological driver is the uneven or excessive release of prostaglandins from the endometrium during menstruation; higher prostaglandin levels correlate with more severe menstrual pain [8, 9]. Despite its prevalence and functional impact, treatment is often suboptimal.

Several approaches have been proposed for pain management, including specific classes of analgesics, hormonal contraceptives, and therapies that inhibit prostaglandin production. Interest in medicinal herbs has grown recently, driven by concerns about pharmaceutical side effects, the cost of importing raw materials for conventional drugs, and reluctance among many young women to take hormonal treatments [10]. However, clinical evidence for herbal therapies remains limited. In a 2014 study, Davari and colleagues found that aromatherapy with rosemary and lavender used individually or in combination reduced both the intensity and duration of PD pain, suggesting potential benefit while underscoring the need for further research on efficacy and safety [11].

Substance P (SP) is a neuropeptide that may physiologically inhibit gonadotropin‐releasing hormone [12]. SP stimulates smooth muscle in the gastrointestinal and urogenital systems and induces vasodilation in blood vessels [13]. In animal models, SP has been identified in the hypothalamic–pituitary axis, uterus, fallopian tubes, and ovaries [13], and it has also been isolated from human ovaries, with primary ovarian carcinoid containing high concentrations [14]. Tachykinins are highly active in the fallopian tubes; exposure increases tubal motility, and it has been proposed that the ovulation‐related rise in motility may result from local tachykinin release [15].

SP and its principal receptor, neurokinin‐1 (NK1R), are implicated in symptoms of chronic and neuropathic pain. The binding of SP to NK1R and tyrosine kinase B receptors contributes to hyperalgesia and central sensitization [16]. Notably, SP appears to hinder the conversion of progesterone to neuroactive gestagen derivatives via NK1R‐mediated mechanisms [17]. SP is released from central and peripheral terminals of small to medium peptidergic dorsal root ganglion (DRG) neurons in response to noxious stimuli. In the dorsal horn, SP enhances synaptic transmission; in peripheral tissues, it triggers neurogenic inflammation. Concurrent blockade of SP at central and peripheral DRG sites may therefore produce antinociception [18].

Nociceptive modulation occurs predominantly in laminae I and II of the dorsal horn, were primary nociceptor afferents synapse with interneurons and projection neurons. Noxious stimulation elicits the release of neuropeptides such as SP, calcitonin gene‐related peptide (CGRP), and somatostatin. These regions also exhibit a high density of glucocorticoid receptors alongside SP and CGRP [19].

NK1R antagonists—such as aprepitant and dexamethasone—target NK1R, which is involved in pain signaling. Although aprepitant is well known for preventing chemotherapy‐induced nausea and vomiting, emerging evidence suggests potential utility in pain management by blocking SP binding to NK1R and disrupting associated signaling pathways. Given its distinct structure relative to other NK1R antagonists, such as netupitant, aprepitant may offer specific advantages in modulating receptor interactions and SP signaling. Also, dexamethasone provides noninferior antiemetic effect via NK1R blockade, if not directly and have been combined with aprepitant in several studies [20, 21]. The multifaceted role of NK1R antagonists in neurokinin pathway modulation provides a rationale for further research and the development of innovative analgesic strategies [22].

On this basis, our study was designed—to our knowledge for the first time—to evaluate the effects of nonsteroidal anti‐inflammatory drugs (NSAIDs) and NK1R antagonists (dexamethasone plus aprepitant) on dysmenorrhea and its associated symptoms.

## 2 Materials and Methods

### 2.1 Study Design and Participants

This pilot randomized controlled trial (NCT06317064, registered at ClinicalTrials. gov) was conducted from March 2024 to December 2024. A convenience sample of 40 female participants was enrolled. Eligible participants were randomly assigned using a computer‐generated block randomization list (block sizes 4 and 6) prepared by an independent statistician. Allocation was concealed with sequentially numbered, opaque, sealed envelopes (SNOSE), opened only after enrollment. Ten females without dysmenorrhea served as controls, and 30 with dysmenorrhea served as cases. The allocation sequence was generated by the statistician, participants were enrolled by the study coordinator, and interventions were assigned by the researcher. Blood was collected across three consecutive menstrual cycles in the case group.

### 2.2 Intervention Sequence (Within‐Participant Across Cycles for Cases)

- **Arm 1:** First menstrual cycle—no medication; blood sampling only.
- **Arm 2:** Second menstrual cycle—NSAIDs—Ibuprofen (400 mg) administered; blood sampling.
- **Arm 3:** Third menstrual cycle—dexamethasone (6 mg) plus aprepitant (80 mg) given for 2 days; blood sampling.

### 2.3 Sample Size

**Figure 1:**
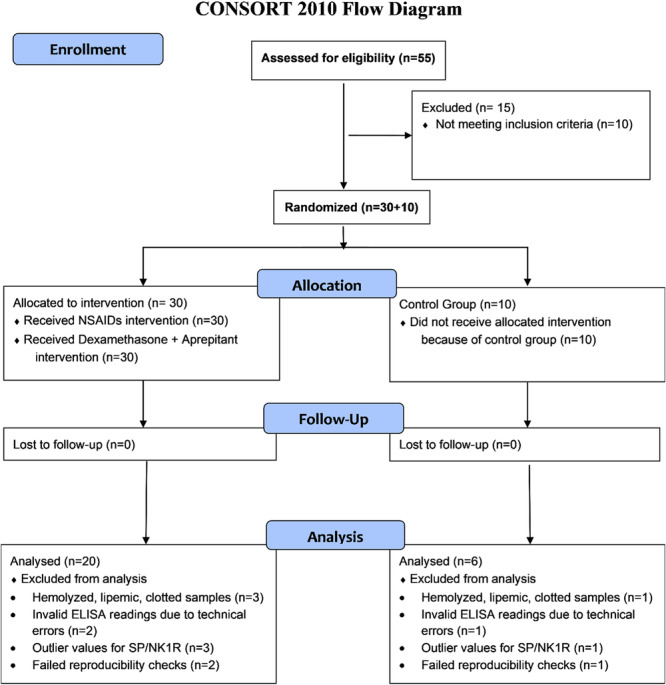
CONSORT diagram of study design for SP and NK1R analysis.

Thirty cases and ten controls were determined a priori to provide 80% power to detect a large effect size (*d* = 0.8, *α *= 0.05). The smaller control group was considered sufficient for this preliminary pilot study, though it limits subgroup analyses, and larger, balanced samples are recommended for future validation. Serum SP/NK1R levels were analyzed in 20 dysmenorrhea cases and 6 controls; 10 cases and 4 controls were excluded due to hemolyzed or lipemic samples, assay errors, contamination, biologically inconsistent outliers, or samples failing reproducibility criteria (Figure 1).

A total of 55 participants were assessed for eligibility, 15 were excluded, and 40 were randomized (30 dysmenorrhea cases, 10 controls). All 30 cases received interventions across three menstrual cycles. Twenty cases and six controls were included in the final analysis after exclusions due to hemolyzed/lipemic samples, technical errors, outlier values, or failed reproducibility checks (Figure 1).

### 2.4 Ethical Approval and Informed Consent

Written informed consent was obtained from all participants after explaining the study purpose, procedures, risks, and benefits. Participants were informed of their right to withdraw at any time. The protocol was approved by the Institutional Review Board of Lahore Medical and Research Center (LMRC/2022/20/RCT003) on December 20, 2022, in accordance with the Declaration of Helsinki.

### 2.5 Eligibility Criteria

Participants were unmarried women of reproductive age. Cases had moderate to severe dysmenorrhea occurring in most menstrual cycles; controls were unmarried women of reproductive age without dysmenorrhea. Exclusions included irregular cycles; current use of contraceptive pills or devices; amenorrhea (menopause or surgical removal); pregnancy or breastfeeding; use of sedatives or NSAIDs; polycystic ovary syndrome (PCOS); prior hysterectomy; and uterine carcinoma. The same exclusion criteria applied to controls.

### 2.6 Data Collection

**Figure 2:**
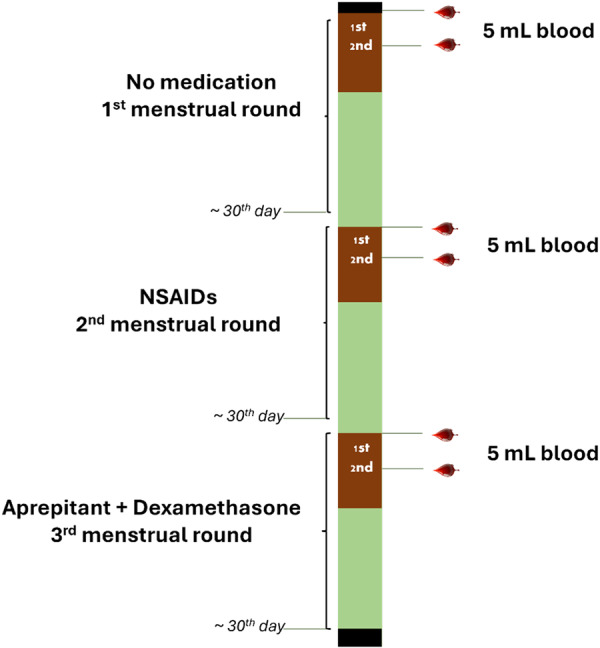
Schematic diagram illustrating data collection strategy.

On Day 2 of the menstrual cycle, 5 mL blood sample was collected before and after each intervention across the three study arms. Complete blood counts (CBC) were performed pre and postintervention to detect hematologic changes. Serum was isolated from all collected samples for downstream analyses (Figure 2). Participants were asked to report any unusual symptoms for safety monitoring. Specialized doctors were part of this monitoring team.

### 2.7 Measurement of Human SP and NK1R

Serum SP and NK1R were quantified using an ELISA kit (BT Lab Human Substance P and Human NK1R kits; Catalogue E1528Hu and E6938Hu). Manufacturer′s instructions were followed, and it was read at a wavelength of 450 nm with automatic readers. Results were adjusted to follow standard curve, and values were reported in picograms per milliliters (pg/mL) for SP and nanogram per milli liters (ng/mL) for NK1R. Each assay was performed in triplicate to evaluate SP/NK1R levels before and after interventions across cycles.

### 2.8 Assessment Tools

#### 2.8.1 Questionnaires

A structured questionnaire captured sociodemographic and clinical parameters, including BMI, family history of pain, age at menarche, cycle length, age of dysmenorrhea onset, and sleep duration.

#### 2.8.2 Visual Analogue Scale (VAS)

Pain intensity was assessed using a 0–10 VAS, where 10 indicates the worst imaginable pain. Based on VAS scores recorded over the first three consecutive days of each menstrual cycle through self‐reporting, participants were categorized as having mild, moderate, or severe pain [23, 24].

#### 2.8.3 Pictorial Blood Loss Assessment Chart (PBAC)

The PBAC—a self‐administered pictorial tool—was used to estimate menstrual blood loss by scoring staining on cotton‐based sanitary pads and tampons across a cycle. Reliability of the PBAC has been demonstrated [25, 26]. Participants received standardized instructions from expert enumerators. They were trained by first showing them sample pictures and analyzing their interpreted scoring. They were guided and corrected for reliable reporting. Enumerators were different from data analysts. PBAC scores ≤ 10 indicated hypomenorrhea, 10–99 indicated normal flow, and ≥ 100 (for 2 months) indicated heavy menstrual bleeding [27, 28, 29, 30]. Cotton pads were the only sanitary products used.

### 2.9 Statistical Analysis

All statistical analyses were performed using GraphPad Prism 8.0.2. Data from questionnaires, CBC parameters, and serum SP/NK1R levels were analyzed to evaluate the effects of the intervention on menstrual pain and related outcomes. Two‐way repeated measures ANOVA was used to assess differences over time and between groups. Assumptions of normality and sphericity were evaluated before analysis. Covariates included in the models were selected based on their known clinical relevance to menstrual pain and related biochemical parameters. Effect sizes were calculated as partial eta‐squared (*η*²) for ANOVA, and 95% confidence intervals (CIs) were reported where appropriate. Statistical significance was set at *p* ≤ 0.05.

### 2.10 Outcomes

The primary outcomes of this study were serum SP and NK1R levels. These were quantified using standardized ELISA kits (BT Lab Human Substance P and Human NK1R kits; Catalogue E1528Hu and E6938Hu) and reported in pg/mL for SP and ng/mL for NK1R. Blood samples were collected on Day 2 of each menstrual cycle, both before and after each intervention across the three study phases. All assays were performed in triplicate to ensure reproducibility.

Secondary outcomes included pain intensity, measured using a 0–10 VAS, self‐reported over the first 3 days of each menstrual cycle, and menstrual blood loss, assessed with the PBAC, a validated self‐administered tool. Hematologic changes, evaluated via CBC, were also monitored for safety. All outcomes measured in the Methods correspond to those reported in the Results section.

### 2.11 Blinding

The allocation sequence was generated by an independent statistician and concealed using sealed envelopes. The researcher analyzing SP/NK1R levels and the data analysts were blinded to group assignments. Participants and researchers were not blinded due to the nature of the interventions, but objective laboratory outcomes were protected from bias.

### 2.12 Protocol Deviations and Adherence

No protocol deviations occurred during the study. All participants adhered fully to the intervention schedule, completed all planned visits, and followed study procedures as instructed. Compliance was monitored through participant logs and investigator confirmation at each visit.

## 3 Results

### 3.1 Baseline Characteristics of Participants

The mean age of the participants was 25.10 ± 7.604 years, their BMI (kg/m^2^) was 20.47 ± 3.607, their mean sleep time was 6.650 ± 1.609 h per day, their menarche age was 12.90 ± 0.7120 years averagely, their mean age of experiencing dysmenorrhea was 14.90 ± 2.468 years and 4.800 ± 0.9 days was their mean length of menstrual cycle. Among the systemic signs of the participants that were included in this study, 90% had cramping, fatigue, and lower back pain. About 50% of participants had no family history of pain. All data points were normally distributed. No adverse effects emerged during the intervention.

### 3.2 Adverse Events

No adverse events or harms related to the intervention were observed during the study. All participants tolerated the interventions well, and no additional medical care was required.

### 3.3 SP/NK1R Levels and Dysmenorrhea

Patient SP and NK1R levels were elevated over two‐fold compared to controls at phase 1 (1375.32 vs. 617.48 pg/mL). For NK1R, patient levels at phase 1 were approximately 4.5 times higher than controls (15.40 vs. 3.38 ng/mL). A clear downward trend in mean NK1R levels was evident from phase 1 to phase 3 (15.40 → 9.49 → 8.42 ng/mL), suggesting significant biological effect of treatment protocol. While mean SP values fluctuated across phases (decreasing in phase 2 and rising in phase 3). This owes to blockade of NK1 receptor sites by phase 3 antagonist, resulting in saturation of SP concentration, subjecting to rise in phase 3. Very high standard deviations indicate substantial variability in individual SP levels within each patient group. Pain scores were consistently and significantly higher in patients (~ 5.5) than in controls (~ 1.1). Mean VAS score showed a minimal decrease by Phase 3 (4.79). Thus, SP and NK1R values were higher in dysmenhorric females than controls. Additionally, SP/NK1R values was reduced after giving NSAIDs intervention in phase 2 and more significantly reduced in phase 3 intervention of NK1R antagonist (dexamethasone + aprepitant) (*p* value < 0.05) (Table 1).

**Table 1:** SP, NK1R, and VAS levels at phase 1 (before medication), phase 2 (NSAIDs), and phase 3 (dexamethasone + aprepitant), and control group.

Group	Phase	*N*	SP (pg/mL)	NK1R (ng/mL)	VAS (0–10)
			Mean ± SD	Mean ± SD	Mean ± SD
Control	‐‐‐	6	617.48 ± 116.33	3.38 ± 1.94	1.10 ± 1.03
Patient	Phase 1	20	1375.32 ± 902.47	15.40 ± 12.10	5.53 ± 2.44
Patient	Phase 2	20	1023.49 ± 957.30	9.49 ± 10.10	5.74 ± 2.40
Patient	Phase 3	20	1325.46 ± 1264.44	8.42 ± 11.90	4.12 ± 1.95

*Note:* Control group measurements are from a single time point.

Abbreviations: NK1R, neurokinin‐1 receptor; SP, substance P; VAS, visual analogue scale for pain.

Mauchly′s Test of Sphericity to verify repeated measures was also conducted. The *p*‐value of 0.446 was greater than 0.05. Therefore, null hypothesis was accepted, which indicated that the assumption of sphericity, a necessary condition for the validity of a standard repeated‐measures ANOVA, has not been violated. This result validates the use of a standard repeated‐measures ANOVA without any correction (e.g., Greenhouse‐Geisser or Huynh‐Feldt) for analyzing the differences in measurements across the three phases in the patient group.

A linear mixed‐effects model was fitted to assess the change in SP and NK1R levels across three study phases in the patient group, accounting for repeated measures within each subject (Table 2). For NK1R, drops in phase 2 and phase 3 were statistically significant (*p* < 0.001 and *p* = 0.006). Estimated mean SP level at phase 1 is significantly different from zero (*p* < 0.001), confirming the high baseline level of SP, especially when compared to controls. Compared to phase 1, SP levels in Phase 2 decreased by an average of 351.51 pg/mL. Compared to Phase 1, SP levels in Phase 3 (postdexamethasone) decreased by a negligible average of 50.19 pg/mL. Both changes were not statistically significant, with *p*‐values of 0.298 and 0.881, respectively, which may largely be due to the small sample size insufficient for determining any significance.

**Table 2:** Fixed effects of phase on SP and NK1R levels in patients.

Fixed effect term	Estimate (*β*)	Standard error	Degrees of freedom (DF)	*t*‐value	*p*‐value
(Intercept)	1374.89	235.53	38	5.84	< 0.001
Phase 2 versus Phase 1	−351.51	333.09	38	−1.06	0.298
Phase 3 versus Phase 1	−50.19	333.09	38	−0.15	0.881
NK1R					
(Intercept)	1.91	1.05	38	1.82	0.077
Phase 2 versus Phase 1	−3.19	0.55	38	−5.82	< 0.001
Phase 3 versus Phase 1	−1.59	0.55	38	−2.91	0.006

Lastly, standard deviation for random intercept (130.96) of SP is much smaller than the residual standard deviation (1053.32), indicating small natural variation in baseline SP levels between different individuals, and larger variation within subjects meaning that an individual′s SP levels fluctuated considerably from phase to phase. Post‐hoc analysis was conducted to perform pairwise comparisons between the study phases, following the linear mixed‐effects model. The Tukey method was applied to adjust *p*‐values for multiple comparisons and control family‐wise error rate. It also concluded similar results mentioned above.

VAS was used to analyze pain intensity of participants before and after medication during menstrual period days. Two‐way ANOVA test formulated *p*‐value of SP/NK1R 0.1754/0.2017 for phase 1, 0.0337/0.0179 for phase 2, while < 0.0001/0.0028 for phase 3 demonstrated that there is a significant difference among number of patients with respect to pain level before and after medication (Table 3).

**Table 3:** Frequencies of patients according to pain severity (VAS) before and after intervention of medicine with respect to SP and NK1R levels.

	*F* (Mean SD)			
Pain severity	Phase 1	Phase 2	Phase 3	*p*‐value
SP levels				
No pain	0	0	6 (623.0 ± 151.0)	< 0.0001*
Mild	0	6 (589.7 ± 155.7)	18 (550.8 ± 66.49)	< 0.0001*
Moderate	18 (854.3 ± 452.2)	24 (1553 ± 1405)	6 (1616 ± 885.1)	0.102
Severe	12 (2231 ± 1718)	0	0	< 0.0001*
*p*‐value SP	0.1754	0.0337*	< 0.0001*	
NK1R levels				
No pain	0	0	5 (24.38 ± 20.84)	< 0.0001*
Mild	0	0	5 (11.32 ± 7.43)	< 0.0001*
Moderate	10 (16.25 ± 16.72)	15 (16.04 ± 13.26)	10 (0.3905 ± 0.0364)	0.0198*
Severe	10 (8.381 ± 8.552)	5 (0.254 ± 1.98)	0	0.2254
*p*‐value NK1R	0.2017	0.0179*	0.0028*	

* *p*‐value is significant (*p* < 0.05). *p*‐value is with respect to frequencies.

The frequency distribution of PBAC score with mean SP/NK1R levels before and after intervention was analyzed. Two‐way ANOVA calculated *p*‐value of 0.017 in normal period group of PBAC scale showed a significant association of PBAC score with SP levels. SP and NK1R levels reduce drastically in phase 2 and 3 as observed by mean values (Table 4).

**Table 4:** PBAC score frequency of participants in association with SP level before and after intervention.

	PBAC (frequency)	Phase 1	Phase 2	Phase 3	*p*‐value
SP	Heavy periods (15)	1910 ± 1661	2021 ± 1614	985 ± 754.5	0.0982
	Normal periods (15)	899.7 ± 484.7	700 ± 167.0	571.8 ± 102.4	0.017*
NK1R	Heavy periods (15)	5.715 ± 7.889	5.556 ± 7.719	4.034 ± 5.334	0.7606
	Normal periods (5)	32.105 ± 17.59	31.696 ± 16.25	24.38 ± 20.84	0.8029

* *p*‐value is significant (*p* < 0.05).

## 4 Discussion

Findings of this pilot study provide, for the first time to our knowledge, an insight into the therapeutic potential of NK1R antagonists′ intervention, particularly the combination of aprepitant and dexamethasone, in mitigating dysmenorrhea. The three‐phase strategy used in the research design, spanning three menstrual cycles, provided a thorough assessment of the intervention′s effects on SP through its receptor NK1R levels. The study demonstrated that dysmenorrhea patients had greater baseline levels of SP and NK1R, implying a link to pain intensity during menstruation. NSAID treatment reduced SP/NK1R levels slightly, whereas dexamethasone and aprepitant decreased them substantially, indicating improved pain relief. SP/NK1R levels consistently increased during menstruation compared to nonmenstruation. Higher PBAC scores were associated with higher SP levels, indicating that variables such as psychological stress and nutritional status impact SP expression, and that dexamethasone combined with aprepitant may effectively alleviate dysmenorrhea.

In comparison to controls, dysmenorrhic patients had greater levels of SP and NK1R before starting any treatment (Phase 1). Phase 2 NSAID usages was related with a decrease in SP and NK1R levels, suggesting that NSAIDs are effective in reducing neurokinin signaling associated with dysmenorrhea. Additionally, on introducing the combination of NK1R antagonists in Phase 3, the greatest decline in SP and NK1R levels was seen. Our findings are in accord with the findings of previous preclinical animal studies. The effect of SP on endosomal signaling complexes involving the NK1R was investigated by Hegron et al. on mice. Anti‐nociceptive effects of NK1R antagonists were seen in their study [22]. Another preclinical study looked at how SP affects pain after a surgical incision. Using an NK1R antagonist in rats, researchers discovered that intrathecal (spinal) delivery greatly decreased pain behaviors, but intrawound medication just reduced guarding behavior. The combined administration improved pain alleviation. SP levels declined in the spinal cord but increased at the wound site, coupled with inflammatory mediators [18].

Kerdelhué et al., also measured SP plasma levels during the LH pre‐ovulatory surge of human menstrual cycle [29]. In another study, Kerdelhué et al., investigated fluctuations in plasma SP levels as well as effects of a particular SP antagonist of the NK1 receptor on pre‐ovulatory LH and FSH surges and progesterone production in the cycling cynomolgus monkey. Comparing these, results in regard to the SP readings from our study signify the potential function of these chemicals in dysmenorrhea [30]. Doses of NSAIDS, and dexamethasone and aprepitant were selected based on clinical evidence [31, 32, 33, 34]. Treatment continued for 2 days for both phases, and samples were extracted on day second when pain levels reach its peaks [33]. Intervention sequence to crossover 30 patients over three menstrual cycles was adopted with the aim of reducing interindividual variations in SP/NK1R levels [35, 36].

To control effects of inter and intraindividual variability in SP/NK1R levels enforcement of similar diet, sleep patterns, and no other medications, strict sampling on second day of menstruation at same time of day, similar assay handling, equipment, and calibration methods (deviations were dropped as explained in methods), and sharp alignment with exclusion criteria for all participants over period of 3 months were ensured. Although results indicated negligible intervariability, intravariability was still evident. One reason might be sample size not sufficient enough to adjust for within‐subject effects. Since SP/NK1R is labile and sensitive to endogenous sex hormones even on the same cycle day, minor variations in hormone surge or luteal/follicular phase dynamics can cause measurable differences in SP/NK1R concentrations. SP is stress‐responsive neuropeptide that may vary due to day‐to‐day fluctuations in personal stressors that cannot be controlled. Gut microbiota and minor immune fluctuations can cause temporary fluctuations in levels. Lastly, variation in drug metabolism among days can also be a leading cause of fluctuations. Further studies and trials are recommended to analyze SP/NK1R levels by controlling for these variables for enhanced reliability of proposed treatment protocol.

Additionally, after NK1R antagonist medication, a significant decrease in the proportion of patients reporting moderate to severe pain was shown by the VAS examination of pain intensity. This supports the relationship between neurokinin signaling and dysmenorrhea and is consistent with the observed decline in SP and NK1R levels. Many studies have been conducted to investigate the effectiveness of various medicines, including NSAIDs such as Ibuprofen, in relieving menstruation discomfort. In general, NSAIDs have been shown to lower pain intensity and improve symptoms in a wide range of people [30, 37].

Due to the study′s limited sample size, further research is needed to validate and generalize the findings. To ensure the robustness and applicability of the observed trends across demographic groups, future studies must include varied populations. The trial′s short follow‐up raises questions regarding the therapy′s long‐term safety and effectiveness. Future studies should provide longer follow‐up periods to assess side effects, menstrual health delays, and pain relief duration.

## 5 Conclusions

This pilot study offers important new information on the therapeutic role of SP/NK1R and the ability of NK1R antagonists. Dexamethasone and aprepitant, in particular to lessen discomfort related to dysmenorrhea. The results point to a possible direction for further investigation incorporating large sample size and controlling for intra‐individual hormone levels.

## Author Contributions

**Riffat Mehboob:** conceptualization, formal analysis, project administration, writing – original draft, writing – review and editing. **Imran Shahid:** conceptualization, writing – review and editing. **Shimaa Mohammad Yousof:** methodology, validation. **Hisham Nasief:** methodology, validation, writing – review and editing. **Samar N. Ekram:** data curation, investigation, writing – review and editing. **Khalid A. Khadawardi:** investigation, resources, writing – review and editing. **Hisham Nasief:** validation, writing – review and editing. **Abdullah R. Alzahrani:** formal analysis, writing – original draft. **Hafsa Adnan:** visualization, writing – review and editing. **Ahmad Alwazzan:** visualization, writing – review and editing. All authors have read and approved the final version of the manuscript. The corresponding author had full access to all data in this study and takes full responsibility for the integrity of the data and the accuracy of the data analysis.

## Ethics Statement

The study was approved by the Lahore Medical Research Center, Lahore, Pakistan (Ethical Clearance Reference Number: LMRC/001/2024/02/16/01). All participants provided written informed consent before participating. Participants also provided consent for publication.

## Conflicts of Interest

The authors declare that the research was conducted in the absence of any commercial or financial relationships that could be construed as a potential conflict of interest.

## Transparency Statement

The lead author, Riffat Mehboob, and Imran Shahid, affirms that this manuscript is an honest, accurate, and transparent account of the study being reported; that no important aspects of the study have been omitted; and that any discrepancies from the study as planned (and, if relevant, registered) have been explained.

## Data Availability

The data that support the findings of this study are available from the corresponding author upon reasonable request.
